# Linking temporal coordination of hippocampal activity to memory function

**DOI:** 10.3389/fncel.2023.1233849

**Published:** 2023-08-31

**Authors:** Guillaume Etter, James E. Carmichael, Sylvain Williams

**Affiliations:** Department of Psychiatry, Douglas Mental Health Research Institute, McGill University, Montreal, QC, Canada

**Keywords:** hippocampus, medial septum, memory, theta rhythms, phase-precession, plasticity

## Abstract

Oscillations in neural activity are widespread throughout the brain and can be observed at the population level through the local field potential. These rhythmic patterns are associated with cycles of excitability and are thought to coordinate networks of neurons, in turn facilitating effective communication both within local circuits and across brain regions. In the hippocampus, theta rhythms (4–12 Hz) could contribute to several key physiological mechanisms including long-range synchrony, plasticity, and at the behavioral scale, support memory encoding and retrieval. While neurons in the hippocampus appear to be temporally coordinated by theta oscillations, they also tend to fire in sequences that are developmentally preconfigured. Although loss of theta rhythmicity impairs memory, these sequences of spatiotemporal representations persist in conditions of altered hippocampal oscillations. The focus of this review is to disentangle the relative contribution of hippocampal oscillations from single-neuron activity in learning and memory. We first review cellular, anatomical, and physiological mechanisms underlying the generation and maintenance of hippocampal rhythms and how they contribute to memory function. We propose candidate hypotheses for how septohippocampal oscillations could support memory function while not contributing directly to hippocampal sequences. In particular, we explore how theta rhythms could coordinate the integration of upstream signals in the hippocampus to form future decisions, the relevance of such integration to downstream regions, as well as setting the stage for behavioral timescale synaptic plasticity. Finally, we leverage stimulation-based treatment in Alzheimer's disease conditions as an opportunity to assess the sufficiency of hippocampal oscillations for memory function.

## Introduction

**Figure 1:**
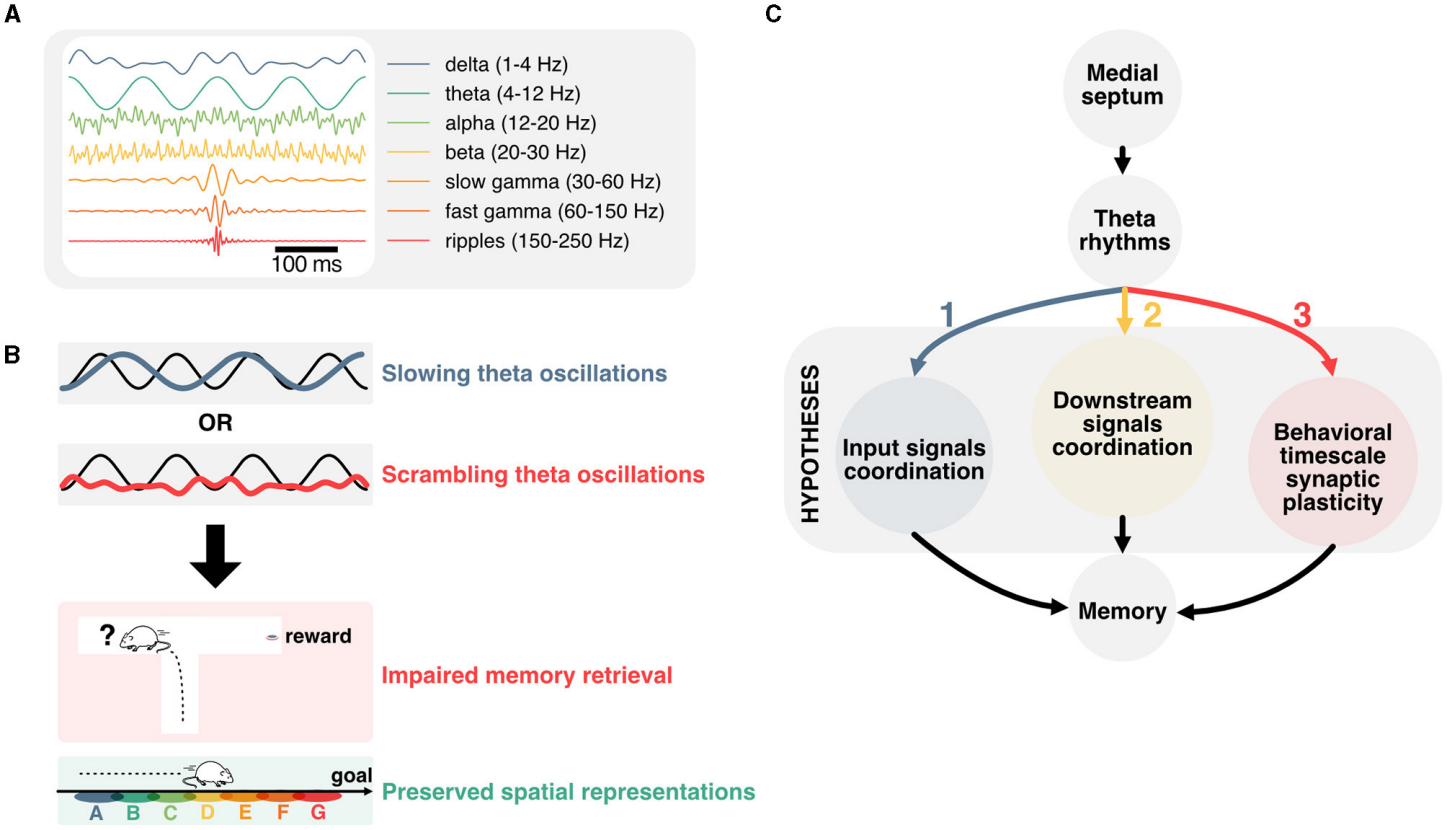
Linking temporal coordination of hippocampal activity to memory function. **(A)** Most prominent hippocampal oscillations and their respective frequency band. **(B)** Disruption of theta oscillations is associated with impaired memory performance, but preserved representations of space. This dissociation prompts to question how theta oscillations contribute to memory function. **(C)** Main hypotheses for how theta oscillations could contribute to memory function. Firstly, hippocampal theta could support the coordination of input signals from upstream regions. Secondly, theta rhythms could contribute to timing hippocampal activities with respect to downstream regions. Lastly, theta oscillations could play an essential role in the temporal coordination of plasticity events, and in particular behavioral timescale plasticity.

One of the most striking features of the hippocampus is its role in encoding and retrieving recent experiences (Scoville and Milner, 1957), providing downstream neocortical circuits with highly associative and context-relevant information (Maviel et al., 2004; Frankland and Bontempi, 2005). In humans, the hippocampus is necessary to imagine the future (Hassabis et al., 2007; Buckner, 2010; Squire et al., 2010; Robinson and Brandon, 2021). This function may be critical in planning actions and learning to predict future outcomes (Keller and Mrsic-Flogel, 2018; Barron et al., 2020; Momennejad, 2020). Neurons in the hippocampus fire in response to task-dependent variables, and in particular spatiotemporal features during active navigation (O'Keefe and Dostrovsky, 1971; Eichenbaum, 2014). The activity of those neurons is organized temporally with respect to oscillations in the theta frequency band (4–12 Hz; Figure 1A) recorded in the local field potential (Buzski et al., 1983; Buzsáki, 2002), which have been described in most mammals (Arnolds et al., 1980; Tesche and Karhu, 2000; Bódizs et al., 2001; Buzsáki, 2002) including humans (Bohbot et al., 2017; Rudoler et al., 2023). These oscillations reflect the sum of transmembrane synaptic currents that synchronize most prominently in the theta frequency band (Buzsáki and Wang, 2012). Although theta oscillations have been proposed to support working memory (Hasselmo, 2005; Fuentemilla et al., 2010; Lisman, 2010) and offline memory consolidation (Poe et al., 2000; Ognjanovski et al., 2014, 2018; Boyce et al., 2016; de Almeida-Filho et al., 2021) the exact underlying mechanisms remain unknown. Neurons in the hippocampus represent past, present, and multiple future trajectories which are segmented in time by individual theta cycles (Johnson and Redish, 2007; Gupta et al., 2012; Wikenheiser and Redish, 2015a; Kay et al., 2020; Zheng et al., 2021). These representations are contingent on salient cues in the environment and may be essential for updating decisions during memory retrieval. Yet, recent data suggest that such representations can be dissociated from local field potential oscillations and memory performance (Petersen and Buzsáki, 2020; Etter et al., 2023) (Figure 1B). This dissociation between the distinct roles of theta oscillations and hippocampal representations in memory is particularly puzzling and will be the focus of this review.

Here, we will first cover landmark studies that established the mechanisms underlying the generation of theta rhythms before reviewing the effects of experimentally disrupting theta on aspects of memory and hippocampal representations. We then explore potential mechanisms that could underlie the disruption of memory encoding, maintenance, and retrieval when controling theta oscillations in spite of hippocampal representations persisting in those conditions. In particular, we look into how theta could coordinate incoming signals to regulate the activity of hippocampal neurons, the implications for downstream regions, and how the interplay between theta rhythms and hard-wired sequential activities could contribute to behavioral timescale synaptic plasticity (Figure 1C).

## Mechanisms for the generation and maintenance of theta rhythms

**Figure 2:**
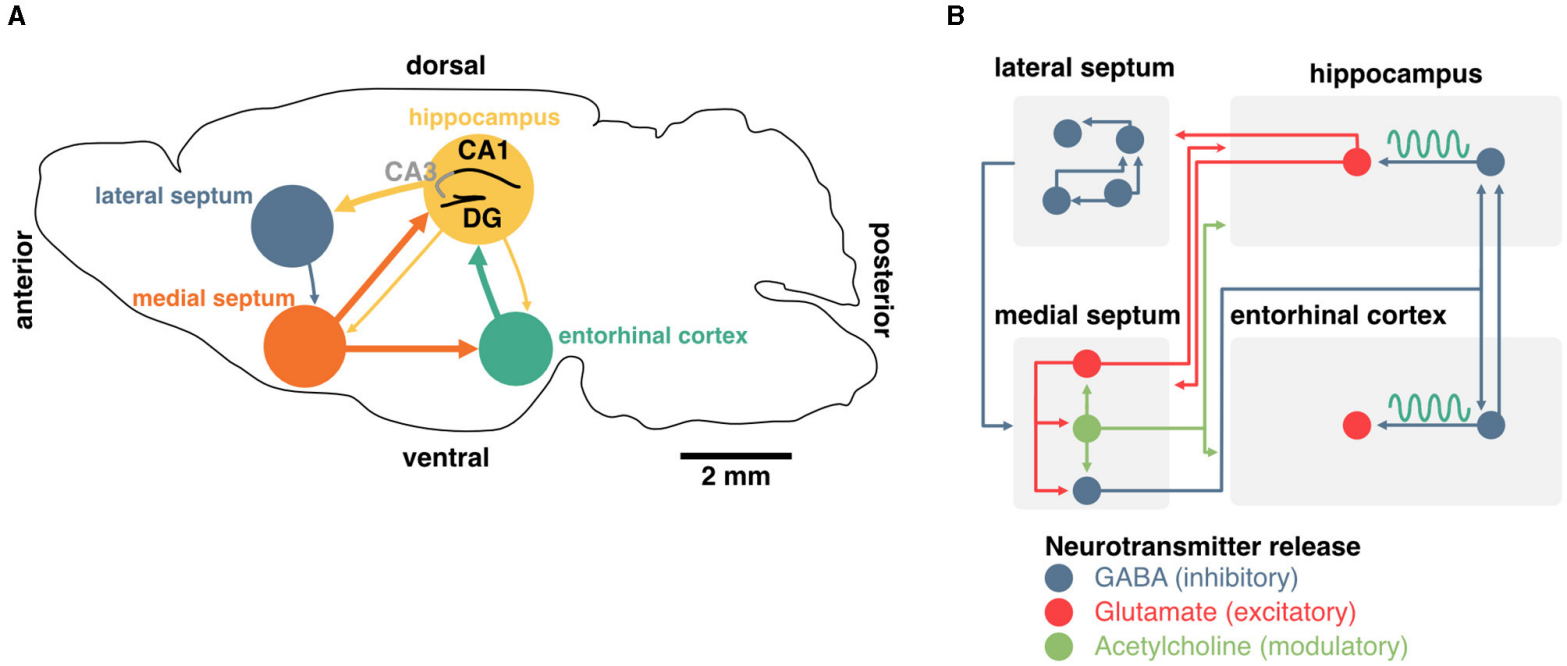
Generation and maintenance of theta oscillations in the septohippocampal system. **(A)** Schema of a coronal section from the mouse brain. Main inputs to the hippocampus originate in the medial septum and entorhinal cortex. The medial septum also projects to the entorhinal cortex, and the hippocampus sends sparse projections to both of these regions. The lateral septum is the most prominent subcortical output of the hippocampus, and sends sparse feedback projections to the medial septum and other subcortical regions. **(B)** Detailed diagram of known connectivity between neurochemically identified cell types in the septohippocampal system that are involved in theta oscillations. In particular, the interaction between septal and hippocampal inhibitory neurons play an essential role in the generation and maintenance of theta oscillations.

The generation and maintenance of hippocampal theta rhythms involve local network interactions as well as external inputs from the medial septum and entorhinal cortex (Figure 2A). In the local hippocampal circuit, theta oscillations can emerge in the absence of external inputs (Goutagny et al., 2009) and rely primarily on interactions between parvalbumin (PV) inhibitory interneurons and excitatory pyramidal cells (Amilhon et al., 2015). Although the hippocampus can oscillate *ex vivo* in the absence of external inputs, lesion or inactivation of neurons in the medial septum lead to a major reduction of theta rhythms *in vivo* (Boyce et al., 2016). This indicates that the medial septum, which provides the main extra-cortical inputs to the hippocampus, contributes to theta oscillations through its interactions with local hippocampal circuits (Rawlins et al., 1979; Mizumori et al., 1990; Jeffery et al., 1995). *In vivo*, neurons in the medial septum fire at theta frequency and this activity likely contributes to driving hippocampal theta rhythms (Freund and Antal, 1988; Paulsen and Moser, 1998; Paulsen and Sejnowski, 2000). While this suggests that the medial septum could act as a pacemaker of hippocampal theta oscillations (Sotty et al., 2003), *ex vivo* recordings of isolated medial septum neurons show that hippocamposeptal activity is capable of driving theta rhythmicity in the medial septum (Manseau et al., 2008).

In addition to the hippocampus, the medial septum also projects to the entorhinal cortex, the main cortical input to the hippocampus. Surgical removal of the entorhinal cortex selectively abolishes theta oscillations corresponding to entorhinal inputs but spares CA1 theta in the proximity of the pyramidal cell layer (Ylinen et al., 1995; Kamondi et al., 1998; Buzsáki, 2002). Altogether, these studies indicate that theta oscillations in the hippocampus emerge through the interplay of local pacemakers and external rhythmic drives including the medial septum.

## Linking theta rhythms to memory function by manipulating the medial septum

Since theta oscillations have been proposed to play a major role in memory function, and the medial septum is essential for maintaining theta drive *in vivo*, a large body of work has focused on establishing a relationship between manipulations of the medial septum, theta physiology, and memory performance. While lesions of the medial septum consistently disrupt or abolish hippocampal theta oscillations (Winson, 1978; Rawlins et al., 1979; Mizumori et al., 1990; Jeffery et al., 1995), targeted circuit manipulations of the medial septum during specific phases of memory tasks have shown a strong correlation between the loss or pacing of hippocampal oscillations and impairments in memory encoding (Gemzik et al., 2021; Quirk et al., 2021; Gonzalez et al., 2022), consolidation (Boyce et al., 2016), reconsolidation (Radiske et al., 2020), and recall (Pastalkova et al., 2008; Wang et al., 2015; Mouchati et al., 2020; Gemzik et al., 2021; Etter et al., 2023) (summarized in Table 1, see Müller and Remy, 2018 for review). Furthermore, stimulating PV interneurons in the hippocampus at either the peak or trough of theta enhanced memory encoding or retrieval, respectively, depending on the stage of a memory task (Siegle and Wilson, 2014), suggesting that coordination between local hippocampal activities and remote septal drive is necessary for memory function.

**Table 1:** Summary of septal and hippocampal manipulations, their effects on hippocampal oscillations and memory function.

**Model**	**Target**	**Manip**.	**θ**	**γ**	**W.M**.	**Enc**.	**Ret**.	**Con**.	**Note**	**References**
Mouse	MS-PV	opto. ~	↑			↓				Quirk et al., 2021
		opto. ↑	⊗		↓	=	↓			Etter et al., 2023
		opto. ~	↑		↓	↓	↓			Etter et al., 2023
	MS-GABA	opto. ↓	↓	=				↓	REM	Boyce et al., 2016
		opto. ↓	↓		=					Yong et al., 2022
	HC	opto. ↑~	=				↑			Rahsepar et al., 2022
	HC-PV	opto. ↑~	↑	↑		↑	↑			Siegle and Wilson, 2014
		pharm. ↓	↓					↓		Ognjanovski et al., 2017
		opto. ↓	↓					↓	NREM	Ognjanovski et al., 2018
		opto. ↑	↑					↑	sleep dep.	Ognjanovski et al., 2018
Rat	MS	Lesion	⊗		↓					Winson, 1978
		Lesion	⊗						↓ learning	Mitchell et al., 1982
		Lesion	⊗		↓	↓				M'Harzi and Jarrard, 1992
		pharm. ↓	↓		↓	↓				Mizumori et al., 1990
		pharm. ↓	↓		↓					McNaughton N. et al., 2006
		elec. ↑~	↑		↑					McNaughton N. et al., 2006
		pharm. ↓	↓	=			↓			Shirvalkar et al., 2010
		elec. ↑	↑				↑			Shirvalkar et al., 2010
		pharm. ↓	↓		↓					Wang et al., 2015
		cool.	↓		↓					Petersen and Buzsáki, 2020
		opto. ↑~	↑		↑					Blumberg et al., 2016
		opto. ↑~	=			=	=			Mouchati et al., 2020
		opto. ↓	↓			=	=		↓ ReCon.	Radiske et al., 2020
		pharm. ↓	↓		↓					Givens and Olton, 1990
		pharm. ↓	↓		↓	=	=			Givens and Olton, 1994
		pharm. ↓	↓	↓	↓					Bolding et al., 2020
	MS-GABA	pharm. ↓	↓	↓	↓					Bolding et al., 2020
		pharm. ↑	↓	↓	↓					Bolding et al., 2020
		pharm. ↑	↓		↓					Givens and Olton, 1990
		pharm. ↑	↓		↓	=	=			Givens and Olton, 1994
	MS-Ach	pharm. ↓	↓		↓					Givens and Olton, 1990
		pharm. ↓	↓		↓	=	=			Givens and Olton, 1994
		pharm. ↑	=		=					Givens and Olton, 1990
	HC	elec. ↑~	↑			↓				Lipponen et al., 2012

θ theta; γ gamma; W.M., working memory; Enc., encoding; Ret., retrieval; Con., consolidation; ReCon., reconsolidation; MS, medial septum; HC, hippocampus; PV, parvalbumin; Ach, acetylcholine; opto., optogenetic; elec., electrical; pharm., pharmacological; fnx, fornix; REM, rapid eye movement sleep; NREM, non-REM sleep; ↑ increased/excitation; ↓ decreased/inhibition; ⊗ abolished/scrambled; ~ pacing; = unaltered.

Although silencing the medial septum as a whole is a crucial starting point in understanding the role of hippocampal oscillations in memory, it should be noted that the medial septum contains three major classes of neurons that have been categorized on the basis of their neurochemical profiles: GABAergic, cholinergic, and glutamatergic neurons (Figure 2B). Non-specific manipulation of septal neurons could therefore lead to side effects due to the confounding release of neuromodulators. In recent years, optogenetic manipulation of targeted sub-populations of septal neurons has shed light on their contribution to hippocampal theta and memory.

Septal neurons that release the inhibitory neurotransmitter GABA might be the most critical in driving hippocampal rhythms. In addition to expressing the vesicular GABA transporter (VGAT) which can be used to target these cells specifically (Boyce et al., 2016), the majority of these neurons express the calcium-binding protein parvalbumin (PV), which is typically found in fast-spiking interneurons (Borhegyi et al., 2004). This population of septal PV neurons targets interneurons in the hippocampus (Freund and Antal, 1988) and entorhinal cortex (Gonzalez-Sulser et al., 2014), a large portion of which also expresses PV (Unal et al., 2015). Theta oscillations can be controlled by this feedforward inhibitory circuit, and rhythmic optogenetic activation of septal PV neurons effectively entrains hippocampal oscillations (Bender et al., 2015; Zutshi et al., 2018; Etter et al., 2019).

The medial septum also contains glutamatergic neurons that specifically express the vesicular glutamate transporter VGlut2 and make up approximately a quarter of all neurons in the region (Sotty et al., 2003; Colom et al., 2005). Septal glutamatergic neurons are largely connected with other septal sub-populations (GABAergic and cholinergic, which we will review next) and display rhythmic activity in the theta band (Manseau et al., 2005). These neurons exhibit diverse firing patterns (slow, fast, and bursting) and have the potential for driving hippocampal neurons in the theta band (Huh et al., 2010). While septal glutamatergic neurons may be critical for spatial memory retrieval (Bott et al., 2022), this control could depend on local interactions with PV neurons (Manseau et al., 2005; Robinson et al., 2016) or by modulating CA3 and CA1 interneurons (Fuhrmann et al., 2015; Robinson et al., 2016).

Finally, the medial septum contains slow-firing neurons that release the neuromodulator acetylcholine. These neurons represent about two-thirds of septal projections to the hippocampus (Sotty et al., 2003; Sun et al., 2014). Optogenetic stimulation of these septal cholinergic neurons has a striking effect of blocking sharp-wave ripples (Vandecasteele et al., 2014) and could have some implications for spatial representations of hippocampal neurons (Mamad et al., 2015). However, cholinergic neurons often co-transmit GABA (Ren et al., 2011; Saunders et al., 2015; Takács et al., 2018) as well as glutamate (Sotty et al., 2003; Allen et al., 2006). Release of GABA was shown to be sufficient to inhibit the expression of sharp-wave ripples (Takács et al., 2018), further complicating the interpretation of specific manipulation of septal cholinergic cells on memory. Although acetylcholine can modulate the activity of hippocampal pyramidal neurons, they are not believed to control theta oscillations directly (Dannenberg et al., 2015). Acetylcholine has, on its own, many proposed functions for memory which we will not discuss here (see Haam and Yakel, 2017 for review). Nevertheless, it is worth mentioning that this neuromodulator could play an important role in controlling the flow of information between the hippocampus and the entorhinal cortex and could be critical for segregating periods of encoding and consolidation (Hasselmo et al., 1992; Hasselmo and Giocomo, 2006; Lovett-Barron et al., 2014). In summary, while both glutamatergic and cholinergic projections to the hippocampus could control some aspects of memory function, the generation and maintenance of theta rhythms specifically rely on the activity of septal inhibitory neurons, either directly, or indirectly (through local circuit interactions; Figure 2B).

The aforementioned studies show that controlling hippocampal oscillations by way of septal manipulations can be complicated by neurochemical heterogeneity. On the one hand, non-specific activation of septal neurons could be associated with spurious release of glutamate and acetylcholine in the hippocampus. On the other hand, selectively silencing septal inhibitory neurons could lead to altered excitatory/inhibitory balance in the hippocampus. More recently, a further refinement in this type of circuit manipulation has focused on altering the timing of theta oscillations, instead of disrupting circuit activity. These manipulations preserve septal drive and offer possible solutions to study the specific contributions of hippocampal oscillations to memory. A first approach has leveraged thermal cooling of the medial septum, effectively slowing down septal activity and in turn reducing hippocampal theta power and frequency (Petersen and Buzsáki, 2020). In another study, optogenetic stimulations of septal PV neurons were used to scramble or pace theta, but not other oscillation frequencies (Etter et al., 2023). In both studies, manipulating theta physiology led to memory defects, yet the tuning of hippocampal neurons to spatiotemporal features remained unaltered, providing a clear example of a dissociation between theta, memory, and spatial tuning. Spatiotemporal coding has long been proposed to serve as a substrate for memory (Kentros et al., 2004; Eichenbaum, 2014; Moser et al., 2015; Lisman et al., 2017), making this dissociation between hippocampal representations and memory performance perplexing. In the following sections, we will explore potential explanations for this phenomenon and in particular why sequential activities of hippocampal neurons can be uncoupled from theta oscillations.

## Hippocampal sequences can occur in the absence of external stimuli

**Figure 3:**
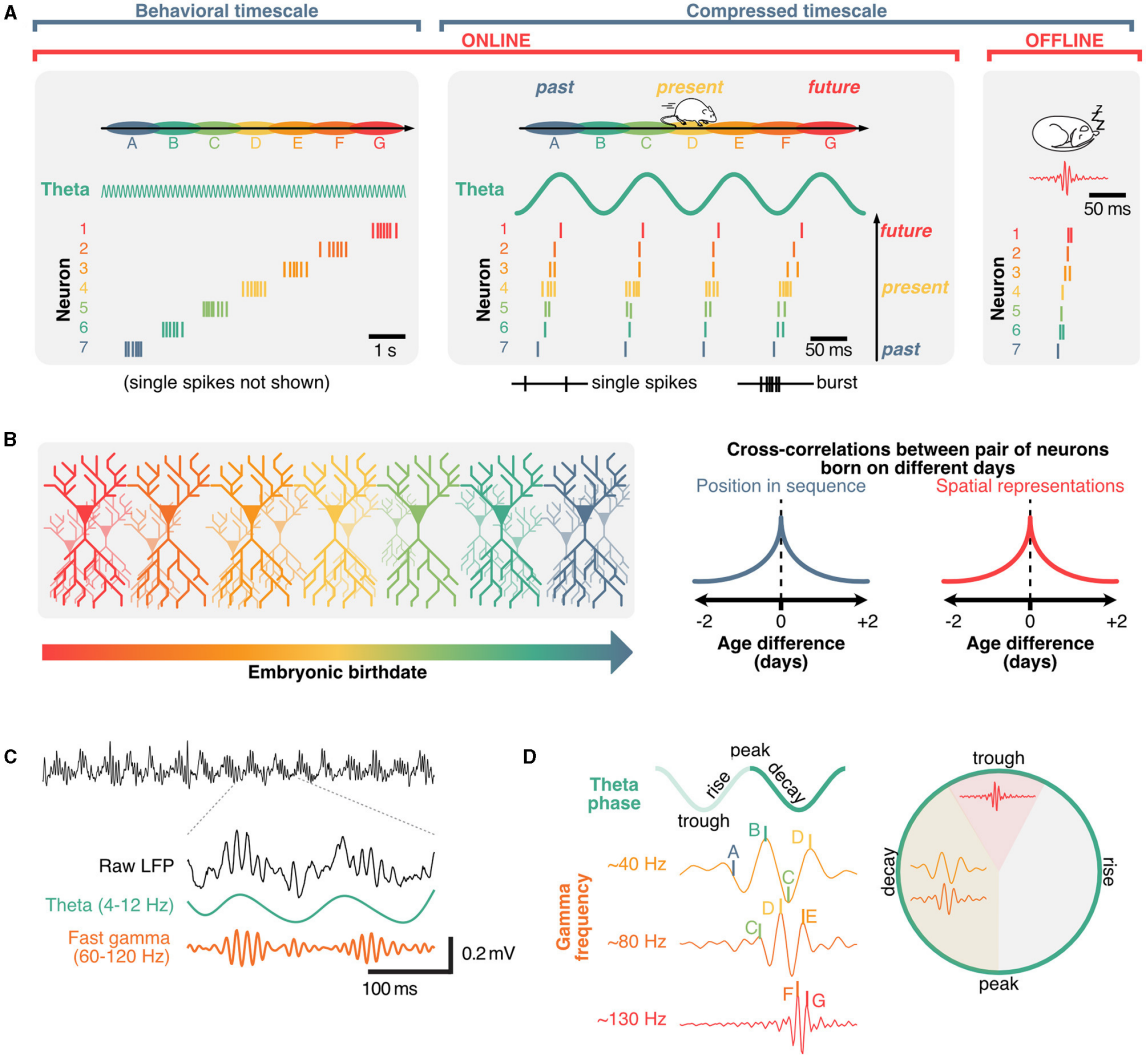
Role of theta and gamma oscillation in coordinating hippocampal sequences into distinct cell assemblies. **(A)** Hippocampal sequences can be expressed in either sub-second, compressed regimes (offline, during sharp-wave ripples or online, in theta sequences) and, when considering faster firing frequencies, on a behavioral timescale as animals explore environments. **(B)** Spatial representations of hippocampal neurons and their position within sequential activities are predominantly determined by connectivity which can be traced back to neuronal birth dates. **(C)** Example raw local field potential trace (black) and corresponding theta (green) and gamma oscillations (orange), which are nest within individual theta cycles. **(D)** Gamma waves further segregate hippocampal sequences into distinct neuronal assemblies, with preferential phase-frequency coupling patterns.

A core property of hippocampal neurons is their propensity to fire in sequences (Buzsáki and Tingley, 2018). On a behavioral timescale (order of seconds), hippocampal sequences can be described regardless of the underlying theta phase and can emerge in the absence of external stimuli (Villette et al., 2015; Haimerl et al., 2019), and typically take the form of relatively fast (~10 Hz), bursting activity (Figure 3A). These activity patterns suggest that the hippocampus uses a primordial neural syntax to represent sequential information over behaviorally relevant periods of time (typically on the order of seconds). Hippocampal sequences could support path integration in the absence of cues (McNaughton et al., 1996; McNaughton B. L. et al., 2006) and anchor to signals from the external world, such as visual cues, when available (Muller and Kubie, 1987; Gothard et al., 1996; Bourboulou et al., 2019). Consistent with this idea of path integration using internally driven self-motion information, hippocampal neurons can represent time (Eichenbaum, 2014) or distance (Ravassard et al., 2013; Aghajan et al., 2015; Etter et al., 2023). Similar sequential patterns are present in hippocampal cells that encode the temporal relationship between events (Manns et al., 2007; Pastalkova et al., 2008; MacDonald et al., 2011). Beyond spatiotemporal variables, recent studies suggest that hippocampal neurons can encode broad, task-specific abstract representations (Nieh et al., 2021). While early interpretations of hippocampal representations have been related to a “cognitive map” that registers the geometry of surroundings (Tolman, 1948), more recent interpretations propose that the hippocampus learns a predictive map instead (Stachenfeld et al., 2017). This model, termed “successor representation,” originates in reinforcement learning (Dayan, 1993; Stachenfeld et al., 2014), and proposes that neurons in the hippocampus represent the value of future states instead of pure spatial locations. This is a reasonable framework as the activity of hippocampal neurons tends not to represent the external world perfectly and instead display activity patterns that are skewed toward their preferred firing location over the course of learning (Mehta et al., 2000) and biases toward rewarding locations (Ormond and O'Keefe, 2022). A key takeaway from the successor representation model is that hippocampal neurons could be involved in predicting future states, which would be particularly relevant for making decisions, to overcome obstacles, or reach goals. Interpreting sequences in the context of successor representations would imply that the value of future states is constantly being evaluated during each theta cycle. The idea that hippocampal neurons might represent future states is further supported by theta sequences and phase-precession.

## Theta sequences support real-time decision-making during memory retrieval

Sequences in the hippocampus can also occur on the scale of milliseconds, and are referred to as “theta sequences” because they are compressed within distinct theta cycles (Skaggs and McNaughton, 1996; Lee and Wilson, 2002; Harris et al., 2003; Diba and Buzsáki, 2007; Pfeiffer and Foster, 2013). While hippocampal place cells tend to burst in their preferred state (“place field”), the activity of other neurons within a theta sequence typically involves single, or a few action potentials and represent past or future states (Dragoi and Buzsáki, 2006; Lisman and Redish, 2009; Figure 3A). Unlike the aforementioned place-cell sequences, these rapid theta-sequences are directly related to the phase of theta, which shift over the course of behavior, a phenomenon referred to as “phase-precession” (O'Keefe and Recce, 1993; Skaggs et al., 1996; Jones and Wilson, 2005; Hafting et al., 2008; van der Meer and Redish, 2011). Additionally, some neurons could represent future possibilities across multiple theta cycles. These neurons, commonly referred to as “splitter cells,” could play a critical role in active inference and decision making (Wood et al., 2000; Ferbinteanu and Shapiro, 2003; Grieves et al., 2016; Duvelle et al., 2023). Given that single action potentials alone are less likely to trigger long-lasting plasticity events by integrating pre-synaptic inputs when compared to bursts (Naud et al., 2022, 2023), sub-second theta sequences could mainly be involved in real-time decision-making during memory retrieval. While the exact role of phase-precession remains to be clearly established, theta sequences can display distinct prospective (ahead of current location) and retrospective (behind) phases, the balance of which depends on behavioral states (Wikenheiser and Redish, 2013) and appears to bias toward behaviorally salient features of the environment or outcomes of decisions (Johnson and Redish, 2007; Gupta et al., 2012; Wikenheiser and Redish, 2015a,b; Kay et al., 2020; Zheng et al., 2021). Sequences representing possible trajectories can be active on alternating theta cycles, a phenomenon called “cycle skipping” (Johnson and Redish, 2007; Kay et al., 2020) which may serve to functionally segregate ensembles within the hippocampus and in downstream regions (Tang et al., 2021). This idea suggests that while sequences could be driven by internal signals, disruptions of theta could in turn cause a mismatch between the ongoing phase or cycle of theta and the timing of neural activities representing possible future states.

If theta rhythms provide a means of segregating multiple representations in time, shifting theta outside of the optimal frequency would result in altered ensemble activity, leading to inaccurate representations and poor memory performance. Septal inactivations that reduce or abolish theta rhythms reliably impair spatial working memory (Table 1) and disrupt theta sequences (Wang et al., 2015), however manipulating the timing of septal and hippocampal activity may offer insights into the specific role of the theta rhythm in ensemble coordination. Pacing the septum and hippocampus within the theta band can enhance or restore working memory performance (McNaughton N. et al., 2006; Shirvalkar et al., 2010; Siegle and Wilson, 2014 but see Etter et al., 2023) while shifting septal activity outside of the endogenous theta range results in memory impairments (Quirk et al., 2021; Etter et al., 2023). Slowing septal drive also produces general working memory impairments which coincide with less compressed theta sequences (Petersen and Buzsáki, 2020). Even with intact theta oscillations, errors during memory recall can be associated with lower theta sequence compression and an underestimation of distances to rewards (Zheng et al., 2021). These less compressed sequences suggest that the disagreement between hippocampal representations of future states and the ongoing theta rhythms may underlie retrieval impairments. Removing septal rhythmicity appears to only impair memory recall during both episodic and working memory (Etter et al., 2023), while experimentally increasing theta frequency has been reported to selectively impair memory encoding only (Quirk et al., 2021).

## Offline, time-compressed sequences reflect preconfigured connectivity

In addition to place cell sequences that occur on a behavioral timescale, and theta sequences that occur on a sub-second timescale, time-compressed sequences can also be expressed offline, beyond periods of active exploration (Figure 3A). These sequences typically coincide with sharp-wave ripples, which are high-frequency oscillations (~200 Hz) that can be recorded in the close proximity of hippocampal pyramidal neurons during quiet wakefulness and non-REM sleep (Wikenheiser and Redish, 2015a; Figure 1A). Having been extensively described (see Buzsáki, 1996, 2015; Liu et al., 2022 for reviews), we will not cover sharp-wave ripple sequences in detail here. It should be noted, however, that the sequential activity of neurons during sharp-wave ripples could reflect some preconfigured connectivity between neuronal ensembles (Huszár et al., 2022). One particular observation supports this idea: while time-compressed sequences have long been thought to represent previous experiences (Foster and Wilson, 2006), sequences of place cells appear to exist prior to a novel experience (Dragoi and Tonegawa, 2011; Farooq and Dragoi, 2019) (although challenged by Silva et al., 2015) suggesting that hippocampal sequences may be—at least in part—dictated by developmental priors.

## Role of structural priors on hippocampal sequences

Given the emergence of online and offline sequences in the hippocampus prior to, or even without receiving sensory information (Dragoi and Tonegawa, 2011; Ólafsdóttir et al., 2015; Villette et al., 2015; Farooq and Dragoi, 2019) there would need to be inherent biases within the hippocampus that would predetermine how new sensory information would be indexed by an existing sequence. This idea is consistent with the fact that the embryonic “birthday” of neurons dictates their topographical location across the septotemporal and radial axes of the hippocampus, which in turn will bias the genetic profile, physiology, connectivity, and representations of space across neurons (Bayer, 1980; Lein et al., 2007; Henriksen et al., 2010; Mizuseki et al., 2011; Valero et al., 2015; Cembrowski et al., 2016; Danielson et al., 2016; English et al., 2017; Masurkar et al., 2017, 2020; Cossart and Khazipov, 2022). In agreement with this idea, neurons born around the same time tend to fire simultaneously within theta cycles and sharp-wave ripples across the sleep/wake cycle, have overlapping spatial representations across environments, and are more likely to belong to the same cell assembly (Huszár et al., 2022; Figure 3B). Strikingly, these structural priors provide a convincing explanation as to why artificially inducing place fields tend to bias onto pre-existing networks in CA1 (McKenzie et al., 2021). These structural priors could also explain why in pathological conditions including Alzheimer's disease, hippocampal sequences can become rigid and not update across environments compared to healthy controls (Cheng and Ji, 2013). Interestingly, theta sequences are resilient to septal inactivation when environmental cues are rich but lose stability when cues are limited, suggesting that the septal theta drive may provide a mechanism for maintaining internal priors in the face of uncertainty (Wang et al., 2016). The idea that at least a portion of hippocampal representations are dictated by developmental priors provides some explanation as to why they can persist in the absence of theta coordination. In this context, how disrupting theta can cause memory impairments remains a critical question. In the following section, we provide an interpretation based on the idea of coordinating pre-synaptic inputs in memory tasks.

## Gamma oscillations could facilitate communication between presynaptic regions and hippocampal cell assemblies

In addition to containing distinct cell assemblies, theta cycles also include nested gamma oscillations, which are fast rhythms within the ~30–150 Hz range (Fries, 2009; Colgin and Moser, 2010; Buzsáki and Wang, 2012; Lisman and Jensen, 2013; Figure 1A) and tend to occur in short bursts (Belluscio et al., 2012; Figure 3C). Similarly to theta, gamma oscillations result from interactions between inhibitory and excitatory neurons (Wulff et al., 2009). Several computational models propose mechanisms of interactions between excitatory and inhibitory neurons in the generation of these oscillations (Tiesinga et al., 2001; Tiesinga and Sejnowski, 2009; Aussel et al., 2018). In contrast to theta rhythms, gamma waves would involve localized oscillators and smaller populations of neurons (Csicsvari et al., 2003), leading to the idea of specialized microcircuits (Hasenstaub et al., 2005) that could co-exist on top of the slower network oscillations. Gamma rhythms can also emerge in isolated hippocampal *in vitro* preparations (Jackson et al., 2011), and optogenetic frequency scrambling of septal PV neurons specifically disrupts hippocampal theta, but not gamma oscillations, further supporting the idea of localized gamma generators rather than a direct result of septal drive (Etter et al., 2023). Previously, oscillations in the gamma band have been classified by their peak frequency, and preferred theta phase (Lopes-dos Santos et al., 2018; Zhang et al., 2019, see Aguilera et al., 2022; Fernandez-Ruiz et al., 2023 for review). Distinct cell assemblies fire in sequences that tend to associate to different gamma oscillations (Zheng et al., 2016), with theta sequences being more reliably expressed during slow gamma waves (Guardamagna et al., 2023) (Figure 3D). While this segregation of gamma oscillations into a set of few distinct classes could be the result of averaging over many theta cycles, it is noteworthy that gamma oscillations can take any frequency on a given theta cycle (Douchamps et al., 2022).

Gamma-based coherence has been a prominent model for communication across the hippocampal-entorhinal circuit and has classically focused on slow and fast gamma oscillations originating in CA3 and medial entorhinal cortex, respectively. These two distinct gammas are then hypothesized to be integrated into hippocampal CA1 with theta oscillations on a cycle-to-cycle basis (Colgin et al., 2009; Schomburg et al., 2014). This would suggest that theta oscillations in CA1 could serve to partition temporal windows that enable the integration of inputs from these upstream regions using alternating gamma waves (Vinck et al., 2023). However, these models have largely been based on correlations between shifting CA3 and medial entorhinal cortex to CA1 coherence in theta and gamma bands. *In vivo*, excitatory inputs from the entorhinal cortex to the dentate gyrus are most coherent in the theta band, while gamma oscillations would be generated locally from presumed local inhibitory inputs (Pernía-Andrade and Jonas, 2014). This predominance of theta over gamma coherence has also been reported between hippocampal CA1 and the medial entorhinal cortex (Zhou et al., 2022). Another potential pitfall in the communication-through-coherence hypothesis is that theta oscillations harmonics could overlap with higher frequency bands (Czurkó et al., 1999; Terrazas et al., 2005) including slow gamma (Petersen and Buzsáki, 2020). The asymmetry of theta oscillations (Belluscio et al., 2012) can lead to harmonics that extend into the slow gamma range (Scheffer-Teixeira and Tort, 2016), which may lead to a misattribution as to the origin of slow-gamma coherence and the degree of spike modulation in the gamma range during movement (Zhou et al., 2019).

Recently, circuit manipulations of the entorhinal cortex suggest that the lateral and medial subregions provide a mechanism to engage specific downstream cell assemblies through slow- or fast-gamma band timing during specific behaviors (Fernández-Ruiz et al., 2021). Gamma-range communication would be fast, on the orders of tens of milliseconds, yet communication across the hippocampus is much slower at roughly half a theta cycle (~60 ms) (Mizuseki et al., 2009). Under the model proposed by Mizuseki et al. (2009), computations within regions can occur at faster (gamma) timescales while inter-regional communication would occur at the slower theta scale. In this case a loss of theta would effectively break an essential communication channel while preserving other oscillations, a hypothesis supported by scrambling septal theta (Etter et al., 2023). Gamma oscillations still offer a mechanism for entraining specific cell assemblies within a region. CA1 dendrites can act as band-pass filters effectively down-sampling gamma range inputs to the theta timescale (Vaidya and Johnston, 2013), but such mechanisms may still allow for the functional segregation of specific cell assemblies by the phase of theta may still be essential for updating representations in downstream readers (Zutshi et al., 2022), albeit at a slower timescale.

**Figure 4:**
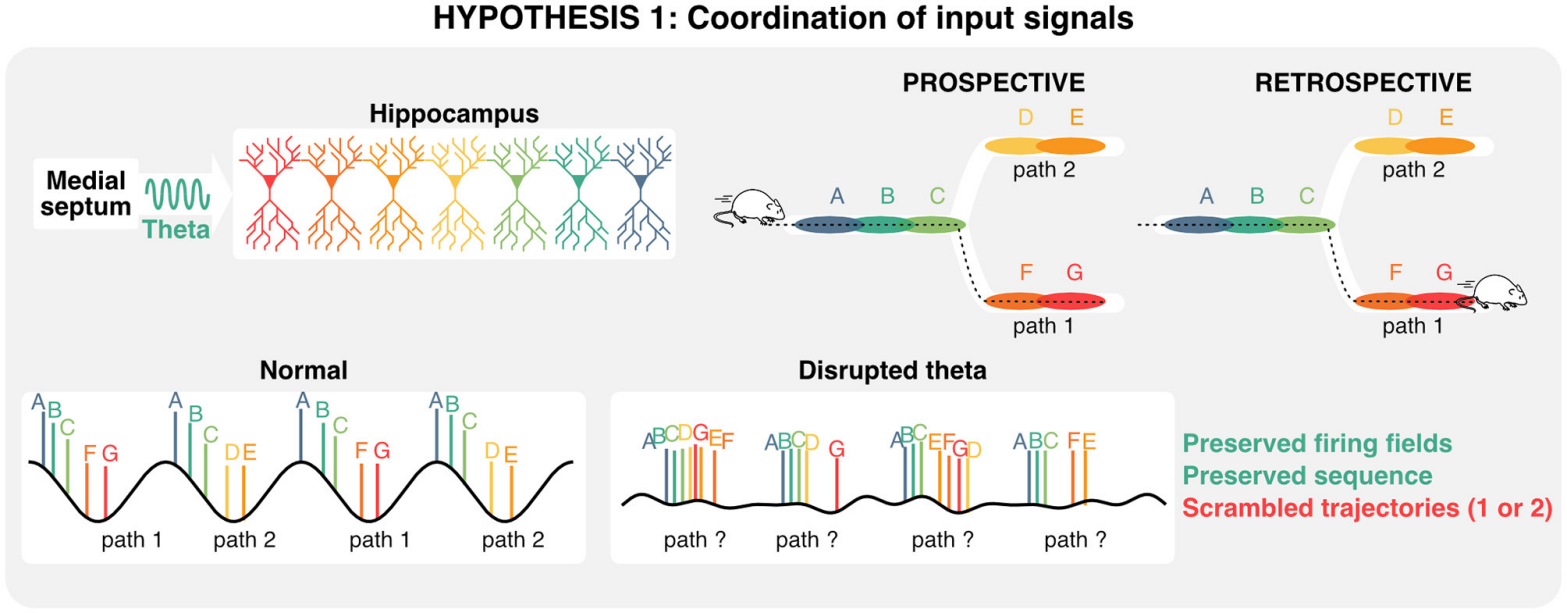
Role of theta oscillation in coordinating inputs during decision making. Given that hippocampal sequences are—at least in part—preconfigured, and that distinct states (each color corresponds to a state) can be represented on alternating theta cycles, disrupting theta oscillations could scramble retrospective and/or prospective trajectories, and in turn impair memory retrieval.

Despite the limitations of gamma communication hypotheses in the hippocampal-entorhinal circuit, it has been proposed that distinct memory functions (encoding vs. retrieval) could be segregated along phases of the theta rhythm and its nested gamma oscillations (Hasselmo and Stern, 2014; Colgin, 2016). Because of its auto-associative properties and role in pattern completion, CA3 would provide signals necessary for memory retrieval during periods of slow (~40 Hz) gamma. In contrast, the entorhinal cortex would provide signals during memory encoding during periods of faster (~80 Hz) gamma (Colgin, 2015a,b). Experimentally, theta phase-specific activation of interneurons during either the encoding or retrieval phase of a memory task produced a small but significant enhancement in performance (Siegle and Wilson, 2014) in line with segmentation models (Hasselmo et al., 2002; Colgin and Moser, 2010; Hasselmo and Stern, 2014). Additionally, slow, but not fast gamma was shown to be recruited at times where memory retrieval is most useful in an avoidance task (Dvorak et al., 2018). Analogous to the aforementioned splitter cells that could represent distinct decisions on individual theta cycles, the idea that distinct gamma oscillations and associated cell assemblies are recruited on separate theta cycles suggests that hippocampal theta oscillations could be computationally useful for segregating and integrating past experiences and future decisions. In summary, hippocampal sequences are at least partly determined by developmental priors, represent past and multiple future states, and coordinate ensemble activity via local gamma oscillations and inter-regional theta rhythms. Consequently, one possible interpretation of the dissociation between hippocampal theta, representations, and memory is that under impaired theta conditions, sequences representing two different trajectories could overlap and confound decision-making (Figure 4).

## Relevance of hippocampal theta for downstream regions

**Figure 5:**
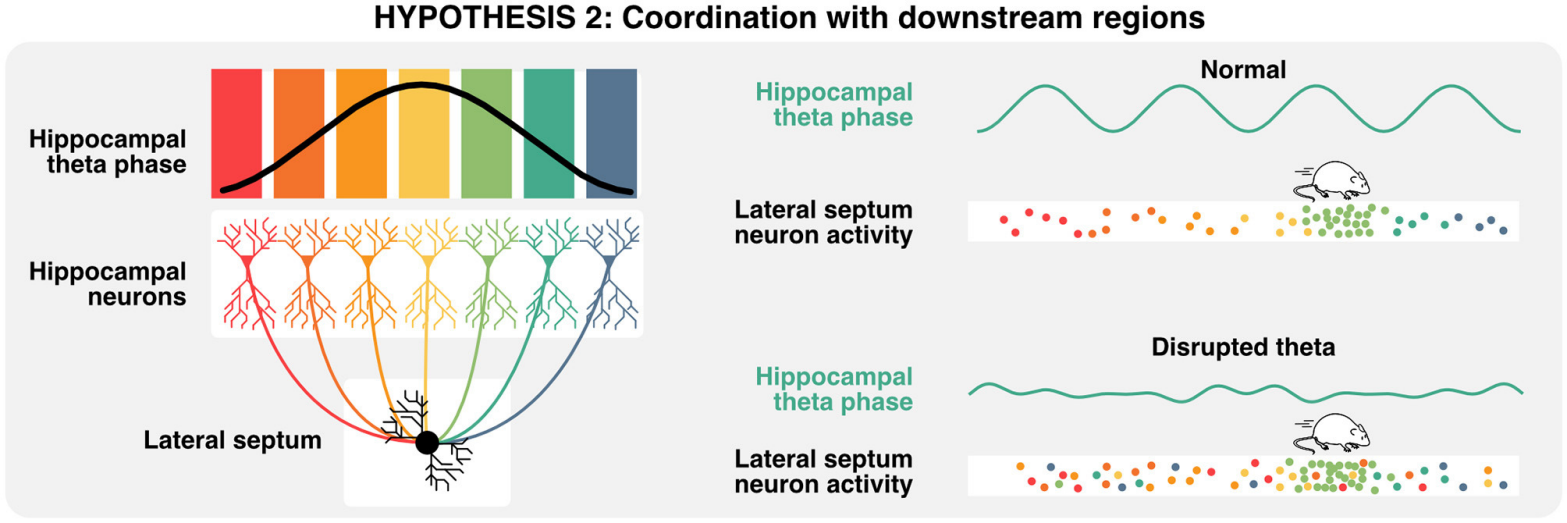
Relevance of theta oscillations for downstream neurons. A single lateral septum neuron can integrate activities from many hippocampal place cells, representing a wide array of states or locations. Lateral septum neurons express preferential activities in a specific location (here in light green), but also encodes multiple spatial locations when considering the phase of hippocampal theta at which single action potentials are emitted. In consequence, the disruption of theta oscillations could lead to the specific loss of phase-place information in lateral septum neurons.

Given that neural computations are highly compositional and increase in complexity and abstraction with every hierarchical level (Hubel and Wiesel, 1962; Moser et al., 2008), hippocampal activities are also relevant in the context of how downstream neurons integrate such signals (Buzsáki and Tingley, 2018). The lateral septum is the most prominent subcortical target of hippocampal projections (Risold and Swanson, 1996; Figure 2A) and contains GABAergic cells that display conjunctive tuning to spatial and self-motion signals (including speed, acceleration, and head direction) similar to CA1 and CA3 pyramidal neurons (Wirtshafter and Wilson, 2019; Veldt et al., 2021). Additionally, the timing of action potentials of lateral septum neurons is locked to specific theta phases at different positions in space, such that the position of the animal can be accurately decoded by the phase at which lateral septum neurons fire (Tingley and Buzsáki, 2018). Furthermore, the functional connectivity between CA3/CA1 and lateral septum (as measured by theta coherence, theta-gamma coupling, and cell assembly strength) shifts from a CA1 bias early on the track to CA3 at later positions and may explain the phase preference of the lateral septum readers. Altogether, these studies suggest that neurons in the lateral septum not only inherit hippocampal representations but also leverage the phase of theta to encode spatial features during active navigation and could be critical for memory retrieval. By extension, one could infer that disruption of theta phase signals would in turn disrupt lateral septum representations of space that specifically depend on the phase of theta (Figure 5). In the following section, we explore how theta rhythms and preconfigured sequences could interact to trigger long-term synaptic plasticity during active exploration and memory tasks.

## Potential roles of theta oscillations in behavioral timescale synaptic plasticity during memory encoding

So far, we have reviewed the potential roles of theta rhythms in memory retrieval, at inference time, from the perspectives of regions that are either upstream or downstream from the hippocampus. As mentioned earlier, retrieval of information occurs on compressed timescales where hippocampal neurons typically emit only a few action potentials within each theta cycle, which are unlikely to trigger long-term plasticity between hippocampal neurons and pre-synaptic regions. Since learning and the acquisition of new information involves long-lasting changes at the level of synapses that either strengthen or weaken communication between neurons, understanding how task-relevant information could be encoded in hippocampal neurons of animals solving memory tasks during learning is essential. While the physiological mechanisms of synaptic plasticity have been well described *in vitro*, the mechanisms through which theta oscillations could orchestrate plasticity events *in vivo* and in real time during learning remain to be defined.

A key signal for synaptic plasticity is the influx of postsynaptic calcium through NMDA receptors and voltage-dependent calcium channels (Lisman, 1989; Nevian and Sakmann, 2006; Graupner and Brunel, 2010; Shouval et al., 2010). To this day, spike-timing-dependent plasticity, which postulates that the precise timing of pre- and post-synaptic activity underlies long-term synaptic plasticity, has been a dominating *in vitro* stimulation protocol (Markram et al., 1997; Sjöström et al., 2001). However, several studies have since challenged the idea that spike-timing-dependent plasticity can form *in vivo* on the basis that it requires a high number of pairings that would be unrealistic for *in vivo* conditions, where online learning occurs on short timescales, and *in vitro* calcium concentrations typically tend to increase synaptic release probabilities beyond physiological levels (Inglebert and Debanne, 2021; Chindemi et al., 2022). While the exact mechanisms of plasticity remain unclear *in vivo*, place cells can emerge *de novo* with only a few excitatory post-synaptic events (Bittner et al., 2015). One mechanism that has been proposed to support this form of behavioral timescale synaptic plasticity is the long-lasting depolarization of post-synaptic neurons (on the order of seconds), a phenomenon referred to as “plateau potentials.” This form of plasticity necessitates pre-synaptic activity *in vivo* (Fan et al., 2023) and can also be replicated experimentally by injecting intracellular current to generate artificial plateau potentials *in vitro* (Bittner et al., 2017). The main idea behind this form of plasticity is that plateau potentials could enable dendrites to “memorize” signals for a longer period of time and support the decoding of temporal sequences (Hawkins and Ahmad, 2016), as demonstrated experimentally (Branco et al., 2010) and with computational models (Leugering et al., 2023).

Given that the ordering of hippocampal sequences is predominantly determined during early post-natal stages and that long-term plasticity can be triggered by only a few action potentials, theta oscillations could play a pivotal role in coordinating these two phenomena (sequences and plateau potentials). This is because theta oscillations are not only observable in the extracellular field potential, but also in intracellular compartments which directly determines excitability, i.e., the degree to which a neuron can be activated by presynaptic inputs. Such excitability varies through time and across cellular compartments of pyramidal neurons including the soma as well as basal and apical dendrites. Calcium can accumulate specifically in apical dendrites (Yuste et al., 1994) that receive prominent inputs from the entorhinal cortex, and concomitant basal and apical activations can drive the generation of plateau potentials reliably (Naud et al., 2023). While the exact effects of theta oscillations on synaptic plasticity remain unclear *in vivo, in vitro* stimulations in the theta band can induce synaptic potentiation in a subpopulation of hippocampal neurons (Sammari et al., 2022).

**Figure 6:**
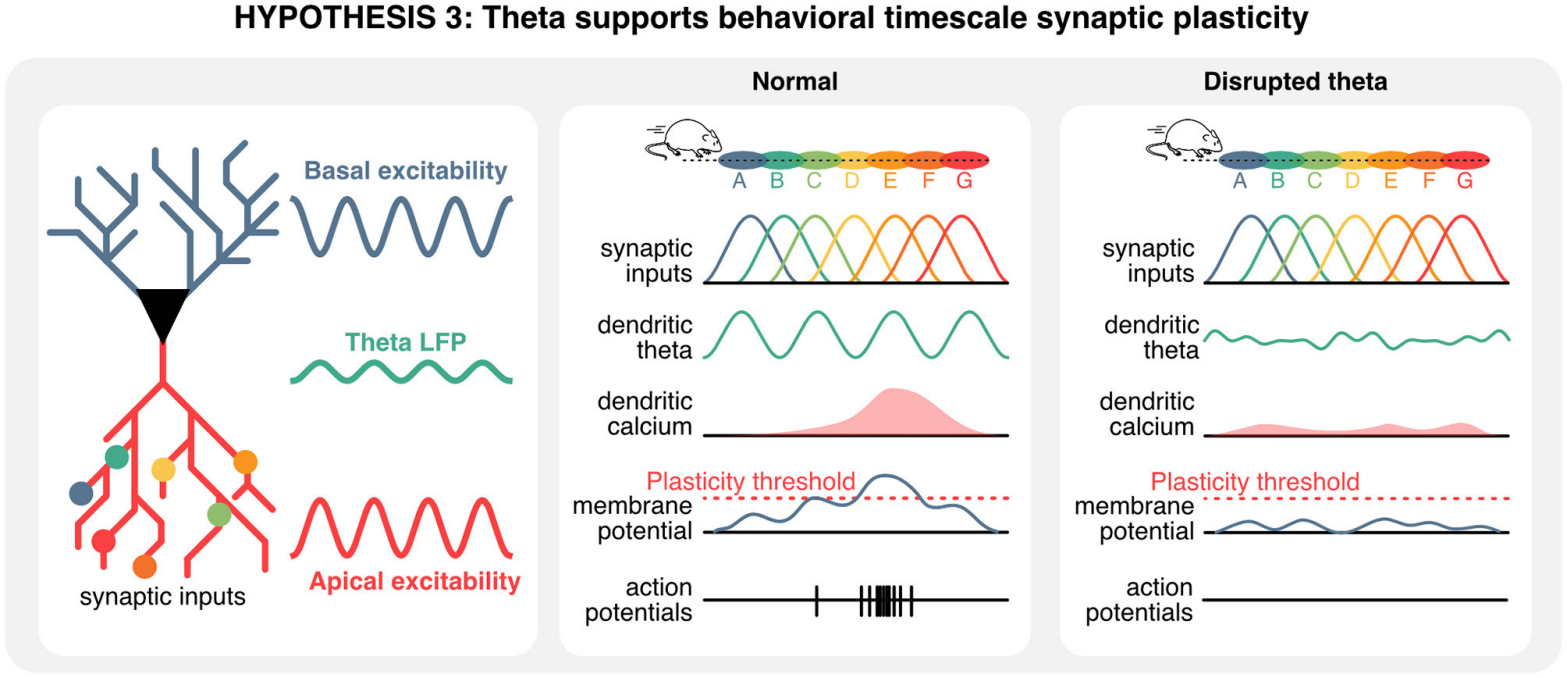
Hippocampal theta oscillations contribute to behavioral timescale plasticity. Hippocampal neurons integrate information from pre-synaptic neurons that represent a wide array of states, including spatial locations. In normal conditions, the timing of pre-synaptic inputs and oscillations of the membrane potential could give rise to plateau potentials. In absence of theta oscillations, a mismatch between hard-wired sequences in pre-synaptic inputs and rhythmic oscillations in the apical compartment could prevent the build-up of plateau potentials, ultimately precluding plasticity, the recruitment of new cell in hippocampal sequences, and thus the encoding of new information.

Taken together, these observations suggest that theta rhythms could play a unique role in setting the stage for behavioral timescale synaptic plasticity, and in turn memory encoding. Considering that a single CA1 hippocampal neuron receives presynaptic information about multiple locations on its dendrites (Sheffield and Dombeck, 2015, 2019), the ablation of theta rhythms could prevent the formation of dendritic plateau potentials necessary for the induction of synaptic plasticity. In these conditions, synaptic inputs and existing sequences would remain unaltered, but the recruitment of *de novo* place cell in existing sequences would be prevented, ultimately disrupting the acquisition of new memories (Figure 6).

## Dissecting space, timing, and memory in Alzheimer's disease

So far, we have leveraged loss-of-function approaches to assess the necessity of theta oscillation in memory. A common phenotype in Alzheimer's disease (AD) patients and animal models is the loss of rhythmicity and cross-frequency coupling which correlate with memory performance. Recently, gain-of-function treatments in neuropathologies, especially in Alzheimer's disease, have focused on restoring network timing in the septohippocampal circuit. AD interventions have attempted to entrain regions or specific populations of neurons to biologically relevant rhythms. As previously discussed, gamma oscillations in the hippocampus are thought to be a marker of healthy circuit function which are linked to specific sequence representations (Zheng et al., 2016). Gamma oscillations are altered in AD patients as well as rodent models (Nakazono et al., 2017; Mably and Colgin, 2018; Jun et al., 2020; Casula et al., 2022) and are correlated with memory impairments (Etter et al., 2019). Focal gamma-band stimulation of hippocampal PV cells has been shown to reduce amyloid beta plaques (Iaccarino et al., 2016) and restore memory performance (Etter et al., 2019) in a mouse model of AD that overexpress the amyloid precursor protein (APP). Auditory and visual stimulation in the gamma frequency has also been studied as a possible non-invasive treatment option with early reports of network entrainment and memory improvement (Clements-Cortes et al., 2016; Iaccarino et al., 2016; McDermott et al., 2018; Jones et al., 2019; Martorell et al., 2019; Adaikkan and Tsai, 2020). However, the efficacy of 40 Hz external light stimulation to entrain native gamma oscillations and remove plaques has recently been called into question (Soula et al., 2023). Nevertheless, optogenetic manipulations provide an excellent opportunity to assess the sufficiency of hippocampal oscillations in memory function and a promising avenue for effective AD treatment (Li et al., 2022).

Coupling between theta and gamma rhythms is a reliable biomarker for healthy hippocampal synchrony and memory in both humans and rodents (Moretti et al., 2009; Tort et al., 2009; Axmacher et al., 2010; Lisman and Jensen, 2013; Heusser et al., 2016; Lega et al., 2016; Vivekananda et al., 2021). Altered theta-gamma coupling strength has commonly been used as a predictor of Alzheimer's disease and has been reported in both APP mice as well as hyperphosphorylated tau mouse models (Booth et al., 2016a,b; Ahnaou et al., 2017; Mably et al., 2017; Mably and Colgin, 2018; Etter et al., 2019; Jun et al., 2020; van den Berg et al., 2023). In an APP AD mouse model altered theta-gamma coupling can emerge before amyloid plaques and hyperphosphorelated tau proteins (Goutagny et al., 2013). Additionally, memory impairments can emerge in young APP mice before plaques accumulate (Francis et al., 2012). These data suggest that altered network timing may trigger a shift in excitation-inhibition balance that could underlie subsequent pathological markers (Goutagny and Krantic, 2013). As discussed above, effective communication in the hippocampus would be best served through an interaction between local ensemble dynamics within the gamma frequency band and the broad theta dynamics. As such, a loss of theta-gamma coupling would mark a failure in effective communication that is required for updating CA1 representations. This raises the promise of targeted early interventions to maintain healthy timing in at-risk brain networks.

Finally, spatial disorientation (Henderson et al., 1989; Tu et al., 2015) and impairments in navigation, which can manifest before the onset of episodic memory loss (Allison et al., 2016), are often overlooked features of preclinical and early onset Alzheimer's disease. Both APP and tau mouse AD models typically display disrupted spatially tuning in grid and place cells in conjunction with navigation or memory impairments (Cacucci et al., 2008; Booth et al., 2016b; Fu et al., 2017; Jun et al., 2020; Ying et al., 2022). Furthermore, AD mice also display inflexible spatial tuning and rigid theta sequences (Cheng and Ji, 2013; Jun et al., 2020) across environments, suggesting that AD model mice may not be able to update representations within the hippocampus. While disruptions of spatial representations is consistently associated with lower memory performance, the link with theta power is unclear. On the one hand, reports differ in the degree of altered theta power in AD conditions, while theta-gamma coupling is more consistently impaired in such conditions (Booth et al., 2016a,b; Ahnaou et al., 2017; Tanninen et al., 2017; Jun et al., 2020; Ying et al., 2022; van den Berg et al., 2023). Together these studies highlight a correlation between spatial tuning and memory, but the functional role of theta appears to be more complicated as in most cases theta rhythms remain unaltered, suggesting that other mechanisms could be involved in memory disruption.

## Summary and perspectives

While hippocampal theta oscillations are essential for memory function, the exact underlying mechanisms remain unclear. Here we summarize landmark studies that defined the mechanisms involved in the generation and maintenance of theta rhythms, which are prominently controlled by medial septum interneurons. This septohippocampal circuit has been specifically targeted to control theta oscillations in health and disease. Importantly, recent studies show that hippocampal theta rhythms and sequences in neural activities can be dissociated. One key takeaway from these studies is that, given the developmental structural priors of hippocampal neurons, their activities with respect to each other might not be relevant for memory function taken out of the oscillatory context. Instead, the timing of those preconfigured ensembles with respect to the phase of theta rhythms could be critical for memory function.

In this review, we propose distinct roles for theta oscillations in the retrieval and encoding of hippocampal-dependent memory. When considering memory retrieval, we propose that theta and gamma oscillations coordinate incoming presynaptic inputs from upstream regions. In this context, disruption of theta rhythms would significantly impact how downstream regions (including the lateral septum) integrate spatiotemporal information. While it was previously proposed that the lateral septum only encodes spatial location through a hippocampal phase code, we propose that this idea could be revised in the light of recent data showing that lateral septum neurons display robust and stable tuning to spatial locations. On the other hand, we also propose that theta oscillations could contribute to the acquisition of novel information during memory encoding. In particular, recent results suggest that plasticity on a behavioral timescale necessitates plateau potentials in the apical compartment of hippocampal pyramidal neurons. Therefore, the timing of preconfigured sequences with respect to the phase of intracellular oscillations would be key in generating such plateau potentials, and in turn, enable learning of new information. Previous work has highlighted the importance of timing pre- and postsynaptic activities for learning. On the other hand, other studies propose that neural oscillations could play a central role in coordinating these pre- and postsynaptic activities. Here we propose that understanding learning *in vivo* requires a holistic view where one must jointly consider synaptic plasticity dynamics, developmental priors in connectivity, as well as oscillations in specific dendritic compartments. Taken together, these hypotheses suggest that the precise coordination of hippocampal neurons would not only be necessary for planning future actions but could also play a key role in supporting plasticity and in turn the recruitment of new neurons in hippocampal sequences. In consequence, we propose testable hypotheses to explain why the disruption of theta oscillations would lead to impaired memory functions. When considering memory encoding, we propose that the precise timing of pre-synaptic inputs and dendritic theta oscillations is necessary for the build-up of plateau potentials necessary to induce long-term potentiation. In absence of these plateau potentials, we predict that pre-synaptic inputs are unable to drive reliable post-synaptic activities and trigger plasticity events. On the other hand, when considering memory retrieval, we propose that the absence of physiological theta could lead to overlap between hippocampal sequences representing different decisions. In these conditions, memory performance would decrease in spite of hippocampal sequences persisting.

Testing these hypotheses experimentally will be key in defining the exact roles of hippocampal theta oscillations in memory function. Since oscillations are generated by the interaction between distinct subpopulations of neurons, disentangling synaptic release from oscillatory activities will be challenging. Nevertheless, such advances will be valuable since manipulations of hippocampal rhythms are already been used as a therapeutic tool for Alzheimer's disease, and would benefit from any mechanistic refinement.

## Author contributions

GE and JC wrote the first version of the manuscript. GE prepared the figures with inputs from all authors. GE, JC, and SW wrote the final version of the manuscript. All authors contributed to the article and approved the submitted version.
